# The impact of theory-based educational intervention on improving helmet use behavior among workers of cement factory, Iran

**DOI:** 10.1186/s42506-018-0001-6

**Published:** 2019-01-07

**Authors:** Hamid Jafaralilou, Iraj Zareban, Mohammad Hajaghazadeh, Habibeh Matin, Alireza Didarloo

**Affiliations:** 1 Health Education and Promotion, Khoy Faculty of Medical Sciences, Khoy, Iran; 2 Health Promotion Research Center, Zahedan University of Medical Sciences, Zahedan, Iran; 3 Department of Occupational Health, Faculty of Health Sciences, Urmia University of Medical Sciences, Urmia, Iran; 4 Health Education and Promotion, Department of Health Sciences, Khoy Faculty of Medical Sciences, Khoy, Iran; 5 Social Determinants of Health Research Center, Department of Public Health, Faculty of Health Sciences, Urmia University of Medical Sciences, P. O. Box: 57561-15111, Urmia, Iran

**Keywords:** Helmet use behavior, Theory of planned behavior, Worker, Cement factory

## Abstract

**Background:**

The occurrence of occupational accidents is a serious public health issue in industrial workers and may impose life jeopardizing complications. The aim of the study was to assess the effect of a training intervention based on the theory of planned behavior (TPB) on helmet use of workers in cement factories in Khoy and Urmia, Northwest of Iran.

**Materials and methods:**

This study was a controlled quasi-experimental investigation (before and after) which was conducted on 170 workers employed in Khoy and Urmia cement factories, Northwest of Iran. Eighty-five eligible subjects from Khoy (as intervention group) and 85 similar samples from Urmia (as control group) were selected and recruited. A valid and reliable four-part questionnaire was used to collect the data including socio demographic information, awareness, the theory constructs, and the behavior. After completing the study questionnaire and needs assessment, a specific educational program was implemented on the intervention group only. The effects of education were compared between the groups before and after intervention. The posttest was applied 1 month after educational intervention.

**Results:**

The mean age of workers in the intervention and control groups was 34.32 ± 8.19 and 33.62 ± 6.17 years, respectively. Before education, the mean score of awareness and helmet use behavior of intervention group was 6.15 ± 3.4 and 5.35 ± 2.8, but after education, those changed into 13.61 ± 3.10 and 9.15 ± 1.65, and the differences were significant (*p* < 0.01). In addition, before education, the mean score of attitude, subjective norm, behavioral control, and behavioral intention of intervention group was 27 ± 5.17, 37.74 ± 6.92, 29.56 ± 6.17, and 17.65 ± 4.90, respectively. After education, the mean score of those changed into 37.26 ± 4.76, 48.34 ± 5.64, 42 ± 8.07, and 24.79 ± 5.33, respectively, and changes were statistically significant (*p* < 0.01), while no statistically significant differences were observed in awareness, the behavior, and the theory constructs in the control group after the intervention.

**Conclusion and recommendations:**

The TPB-based educational approach had a remarkable effect on helmet use of workers. Applying this theory to improve workers’ personal protective behaviors is recommended and emphasized.

## Introduction

Annually, the occurrence of various kinds of occupational accidents in different workplaces imposes various damages to the active manpower and tends to develop life-threatening disabilities and situations [1]. Occupational accident is any physical injury conditions sustained on a worker in connection with the performance of his or her work [2]. In 2016, The International Labor Organization (ILO) reported that there are over 270 million occupational accidents, and those cause two million deaths annually [3]. In many countries, despite the relative improvement in workplace conditions, occupational accidents and its consequences are increasing among employed workers. Studies show that 80–90% of causes of occupational accidents in industrialized countries are due to the unsafe behavior of workers and 10–20% is related to the unsafe working conditions. Although the first priority in the workplace is to reduce hazards through the design and engineering controls, lowering human errors through educating workers and improving their health behaviors are also important [4].

One of the most common human errors is the lack of use of personal protective equipment (PPE). In fact, 34% of occupational accidents were resulting from the lack of use of PPE available at workplace at the time of the accident. In addition, 13% of work-related accidents result from the inappropriate use of PPE [4]. Some studies found that there is a positive relationship between the social and managerial support and the use of PPE at the workplace [5–7].

The PPE refers to protective clothing, helmet, or other equipment used by workers to reduce the specific occupational hazards [8]. In other words, PPE includes a wide range of equipment designed and manufactured to protect the various parts of body (from hair to feet) against a variety of possible risks at the work environments. In fact, PPE prevents from injuries or infections through creating a barrier against risk factors [9]. The head of workers could be injured in working settings such as the cement industry when they are involved in activities such as extraction and crushing, baking, production, packaging, and delivery. Even head damage has been reported in other occupational activities such as packaging, building construction, machinery repair services, warehousing, and welding [9].

Improved workers’ health promoting behaviors can prevent occupational injuries in workplaces. Workplace can be regarded as a suitable environment for training, shaping health protecting behaviors, and implementing screening programs on major group of workers [10]. To obtain useful and efficient results, trainings should be designed based on health education theories and models. These theoretical frameworks provide a systematic view of events or achievements and include a systematic process to analyze the successes or failures. In addition, these approaches, as a way map, provide the required guidance to the educational needs assessment, design, implementation, and evaluation of educational interventions [11, 12]. These approaches have potential to promote workers’ efforts to reduce the unwanted accidents [13].

The theory of planned behavior (TPB), as a behavior change theory, is widely applied to alter and improve different health behaviors such as nutritional behavior of women [11], students’ self-monitoring intention and safe practices in workshops and laboratories [14], the appropriate method of manual load carrying among workers [15], effectiveness of training on the workers’ safety performance [16], and obesity preventive behaviors among high school students [17]. The TPB is an extension of the theory of reasoned action which includes an additional construct called the perceived behavioral control [13]. The TPB assumes that behavioral intention determines behavior directly and indicates that three factors influence an individual’s intention, namely attitudes toward a behavior, subjective norms, and perceived behavioral control (PBC) [18].

When an individual wants to perform a behavior, he/she, first, evaluates the outcome and then forms an intention to perform it. Subjective norms are based on the fact that the individual is influenced by others in the society, including partners, parents, religious leaders, relatives, and health officials, and accordingly performs or avoids performing a behavior under their influence or pressure. In fact, the individual builds his intention based on the demands of others. PBC refers to the degree to which an individual feels that the performing of any given behavior is under his volitional control. If the individual believes that there are no opportunities or resources to perform a particular behavior, he/she is likely not to form a strong intention to perform that behavior even though he/she might have positive attitudes toward it or believe that important others would approve it [12, 17].

Considering that the TPB can help to predict and understand environmental and individual factors affecting behavior, the present study aimed to assess the effect of the TPB-based training intervention on helmet use behavior of workers employed in cement factories located at Khoy and Urmia, Northwest of Iran. The results of this study can be helpful in improving health behaviors, and health of the workforce, in turn, may lead to increase the productivity of production centers.

## Materials and methods

This study was a pretest-posttest quasi-experimental research which was conducted on a sample of workers employed in Khoy and Urmia cement plants, Northwest of Iran, in 2016. Considering the test power of 90%, confidence level of 95%, and using results of the previous study [16], the minimum sample size was calculated to be 57 individuals for each group. Considering the attrition of samples during the study, the final sample size was increased to 85 individuals for each group.

Inclusion criteria were workers living in Khoy and Urmia cities, employment in cement factory, and need to use helmet in the workplace, in 2016. Eighty-five eligible workers were recruited from Khoy cement plant as the intervention group, and 85 eligible samples from Urmia cement factory as a control group. To collect data, a self-report questionnaire including the following parts was used:

1. *Demographic information*: Age, gender, marital status, education level, family income, history of work, and history of injury.
2. *Awareness*: Participants’ awareness was measured through four items about the role of PPE in injury prevention, the benefits of using helmet, characteristics of a safe helmet, and key points in care of a helmet. In this scale, each item had more than one correct answer, and each correct answer was scored 1. The score of items 1 and 2 ranged from 0 to 5 and items 3 and 4 ranged from 0 to 4. Therefore, the total score of awareness ranged from 0 to 18.
3. *The TPB subscales*: The four subscales of the TPB (attitude, subjective norms, perceived behavioral control, and intention) were measured using 49 items:
  The subscale of attitude was examined using nine items based on a 5-point Likert scale (from completely disagree = 1 to completely agree = 5, with the score range of 9–45).
  The subscale of normative norms was assessed by 12 items based on a 5-point Likert scale (from completely disagree = 1 to completely agree = 5, with a score range of 12–60).
  The subscale of the perceived behavioral control was evaluated by 18 items [(13 items about the control beliefs based on a 3-point Likert scale (difficult = 1, neutral = 2, and easy = 3) and five items about the perceived control power based on a 3-point Likert scale (definitely yes = 1, not known = 2 and definitely no = 3, with the score range of 18–54)].
  Finally, the subscale of intention was examined by 10 items using a 3-point Likert scale (definitely no = 1, not known = 2, and definitely yes = 3, with the score range of 10–30). In all subscales of the TPB, we utilized the means of total score to compare between the two groups.
4. *Participants’ helmet use behavior*: It was evaluated by 11 items. Participants were asked to provide answers to items such as the following: Do you use the helmet throughout the working shift? Do you even use the helmet in the absence of an employer? Do you use the helmet despite severe heat and sweating? and so on. Helmet use behavior was rated based on a 2-point Likert scale (yes = 1 and no = 0; with the score range of 0–11).

To assess the content validity of the whole questionnaire and its subscales, the content validity index (CVI) and content validity ratio (CVR) were applied. The study tool was sent via email to 10 academics to act as a panel of experts and examine the items in terms of necessity, relevance, and clarity. Then, the value of CVR and CVI for each item and the whole items of the instrument were calculated. Content validity of the finalized questionnaire was confirmed based on the results of CVR and CVI. The reliability of the whole questionnaire and its domains was also confirmed using value of Cronbach’s alpha. Table 1 presents the detailed information about content validity and reliability of the study instruments.

**Table 1:** Results of validity and reliability of the whole questionnaire and its subscales

Subscales	Item counts	CVI^#^	CVR^##^	Cronbach’s alpha
Awareness	4	0.85	0.78	0.92
Helmet use behavior	11	0.81	0.76	0.97
Behavioral intention	10	0.82	0.78	0.92
Attitude	9	0.83	0.79	0.92
Subjective norm	12	0.83	0.76	0.92
Behavioral control	18	0.81	0.78	0.89
The whole questionnaire	64	0.82	0.77	0.92

^#^Content Validity Index

^##^Content Validity Ratio

Before intervention, the finalized questionnaire was completed by the two groups. Based on the results of questionnaires, the educational package was designed for the intervention group. After coordinating with the managers of cement factory, the training program for the intervention group was held in the training class located at the cement factory. To better implement the educational program, the group was first divided into four equal groups. Then, the training program was presented in two 2-h sessions for each subgroup. In the first session, the educational content including work health and safety, existing risk factors in a cement factory, preventive measures of risks and injuries, and the PPE was presented to subgroups using lectures and question and answer methods. To increase workers’ awareness, educational pamphlets were also distributed among the members of the intervention subgroups at the end of each session.

In the second session, the educational content related to the importance of workers’ health, benefits of using the PPE especially helmet, and the facilitating and inhibiting factors in helmet use behavior was delivered to subgroups through brainstorming and group discussion methods. In educational sessions, normative beliefs and motivation to comply of workers were influenced by using educational techniques such as a role play or psychodrama, and discussion. To modify control beliefs, educator used discussion about factors that facilitate behavior, provide incentives, and reduce inhibiting factors. To impact perceived power, educational methods such as having role models model the desired behavior, removing barriers, and breaking down the behavior into small steps were used. One month after educational intervention, the study questionnaires were re-administered to the two groups. All workers completed the second questionnaire; there were no drop outs.

### Statistical analysis

Data were analyzed using the statistical package for social sciences (SPSS, version 16; SPSS Inc., Chicago, IL, USA). Results of Kolmogorov–Smirnov test showed that only age had normal distribution while data such as knowledge, behavior, and TPB constructs had non-normal distribution. Descriptive statistics such as frequency, percentage, arithmetic mean, and standard deviation (SD) were utilized. Comparison of the data in study groups was done using chi-square test for qualitative variables and independent samples *t* test, and Wilcoxon test for quantitative variables. In this study, a *P* value less than 0.05 was considered significant in all the analyses.

## Results

In the present study, the mean age of workers in the intervention and control groups was 34.32 ± 8.19 and 33.62 ± 6.17 years, respectively. According to the independent *t* test, no significant difference was found between the two groups in terms of age (*P* = 0.53). The results of chi-square test indicated that there was no statistically significant difference between the two groups in terms of marital status, educational level, income level, job experience, and history of the work-related injuries. In addition, all the studied subjects were male. Table 2 presents more details about the socio demographic variables of the studied groups.

**Table 2:** Frequency distribution of demographic characteristics of the intervention and the control groups of workers in two cement factories in Khoy and Urmia, Iran, 2016

Group	Intervention	Control	Chi-square/independent *t* test (*p* value)
	*N* (%)	*N* (%)	
Demographic variables			
Age (years)			0.53
< 30	36(42.4)	31(36.5)	
30–60	49(57.6)	54(63.5)	
Mean age	34.32 ± 8.19	33.62 ± 6.17	
Education level			0.49
Primary school	12(14.1)	11(12.9)	
Middle school	24(28.2)	20(23.5)	
High school	40(47.1)	38(44.7)	
Higher degree of education	9(10.6)	16(17.6)	
Marital status			0.54
Single	22(25.9)	16(18.8)	
Married	63(74.1)	69(81.2)	
Working experience(years)			0.16
< 3	17(20)	15(17.6)	
3–5	32(37.6)	22(25.9)	
> 5	36(42.4)	48(56.5)	
History of injury			0.41
Yes	15(17.6)	15.3	
No	70(82.4)	84.7	
Monthly income (Rials)			0.11
< 15,000,000	67(78.8)	69.4	
≥ 15,000,000	18(21.2)	30.6	

Before the educational intervention, the mean score of the two groups’ awareness about occupational health and safety, PPE, characteristics of a safe helmet, the benefits of using helmet, and the key points in care of a helmet was similar and the same, and no statistically significant difference was found between the two groups in terms of awareness (*p* > 0.05). In addition, the mean score of helmet use behavior in the two groups was similar, and no statistically significant difference was observed between them (*p* > 0.05). After intervention, considerable and significant changes were found between two groups in terms of helmet use, and these changes were confirmed by statistical analyses (*p* < 0.05) (Table 3).

**Table 3:** The mean scores of awareness and behavior of workers in the intervention and control groups before and after the educational Intervention in Khoy and Urmia, Iran, 2016

Group	Intervention			Control		
Variables	Before intervention	After intervention	Wilcoxon (*p* value)	Before intervention	After intervention	Wilcoxon (*p* value)
	Mean ± SD	Mean ± SD		Mean ± SD	Mean ± SD	
Awareness	6.15 ± 3.4	13.61 ± 3.1	0.001*	6.76 ± 2.82	7.13 ± 2.69	0.29
Helmet use behavior	5.35 ± 2.8	9.15 ± 1.65	0.001*	5.18 ± 2.64	5.28 ± 2.45	0.31

*SD* standard deviation

*Significant at *p* < 0.05

Before the educational intervention, the mean scores of behavioral intention, attitude, subjective norm, and behavioral control in the intervention group were 17.65 ± 4.9, 27 ± 5.17, 37.74 ± 6.92, and 29.56 ± 6.17, respectively. After the intervention, the mean score of the above variables significantly increased (*p* < 0.05). In contrast, in the control group, no remarkable and significant changes were found before and after the time of the educational intervention (*p* > 0.05) (Table 4).

**Table 4:** The mean scores of the TPB constructs in the intervention and control groups before and after the educational intervention in Khoy and Urmia, Iran, 2016

Group	Intervention			Control		
Variables	Before intervention	After intervention	Wilcoxon (*p* value)	Before intervention	After intervention	Wilcoxon (*p* value)
	Mean ± SD	Mean ± SD		Mean ± SD	Mean ± SD	
Attitude	27 ± 5.17	37.26 ± 4.76	0.001*	28.28 ± 5.68	29.30 ± 5.50	0.27
Subjective norms	37.74 ± 6.92	48.34 ± 5.64	0.001*	36.14 ± 5.59	35.15 ± 5.54	0.39
Perceived behavioral control	29.56 ± 6.17	42 ± 8.07	0.001*	28.22 ± 5.80	27.29 ± 5.70	0.18
Behavioral intention	17.65 ± 4.90	24.79 ± 5.33	0.008*	18.54 ± 3.90	19.59 ± 3.80	0.15

*SD* standard deviation

*Significant at *p* < 0.05

## Discussion

The purpose of the present study was to evaluate the impact of the TPB-based educational intervention on promoting helmet use behavior among workers employed in two cement plants. Theoretical frameworks can be helpful for health education specialists to identify effective factors on health behaviors and to design appropriate interventions. The impact of educational interventions on human behavior change has been demonstrated by various researchers [19–22]. The findings of the current study were in line with the previous works and showed that the use of the helmet by the workers in the intervention group increased after the intervention.

The study conducted by Adewoye et al. in Nigeria indicated that educational intervention was effective on the use of various PPE, especially helmet in welders [23]. A systematic review of the published documents from 1980 to 2001 revealed that theoretical frameworks such as the TPB, the theory of reasoned action (TRA), and the health belief model (HBM) were effective on prevention of unwanted incidents through improved health behaviors of workers [10, 13].

Lajunen et al. compared the predictive ability of the HBM, TPB, and the Locus of Control (LOC) on cycle helmet use behavior among teenagers. Results of their study indicated that the TPB predicted and explained high percentage of the use of helmet among adolescents compared to the other mentioned theoretical frameworks [24]. In addition, Ross et al. assessed undergraduate helmet use attitudes and behaviors based on the TPB. Their study supported the ability of the TPB in predicting helmet use among college students [25].

The findings of the aforementioned studies were in line with results of the present study. Behavioral changes may be influenced by various factors such as personal awareness and beliefs. In other words, people’s awareness and beliefs have the main role on improving health behaviors. The findings of the present study indicated that the theory-based training led to increase the mean score of awareness and constructs of TPB framework in the intervention group. The study of Navidian et al. in Iran (2014) aimed to assess the impact of safety education on knowledge, attitude, and the use of PPE among workers employed in a glass industry. They found that safety training through motivational interview has resulted in significant difference in the mean score of knowledge, attitude, and behavior of using PPE in the intervention group compared to the control [4].

The survey of Kakaie et al. revealed that the constructs of the belief, attitude, subjective norm, enabling factors (BASNEF) as a behavior change framework were effective in changing the behavior of using personal protective equipment [8]. The results of a study in Malaysia aimed at investigating the intention and behavior of engineering students based on TPB showed that behavioral control and attitude of students compared to subjective norms had high predictive power toward intent to perform safe behaviors in engineering workshops and labs. This study also revealed the relationship of students’ real behavior with their knowledge and behavioral intention [14]. The study conducted by Jafari Kuchi et al. evaluated the effect of TPB on manual material handling, and its results indicated that the TPB framework was effective in changing and improving manual material handling among workers [15].

In addition to the above previous research, other studies of educational intervention based on TPB have proven their effectiveness on different health behaviors such as self-care behavior in women with type 2 diabetes [26], improved preventive behavior of urinary tract infection in pregnant women [27], raising optimism level among high school students [28], and the self-care behaviors for prevention of hypertension among Iranian girl teenagers [29]. The results of the mentioned studies supported and confirmed the findings of our study.

### Study limitations

The absence of some workers in training sessions and difference in the level of workers’ literacy were among the limitations of the present study, though these barriers and limitations were overcome by holding more training sessions and leveling the workers for training. In addition, the data were collected using a self-reported questionnaire. Participants may underestimate or overestimate their own helmet use behavior, which may have affected the study findings.

## Conclusion and recommendations

This study revealed that the educational intervention based on the theory of planned behavior was useful and applicable for modification of workers’ beliefs and improvement of their health behaviors especially helmet use. Using cognition theories/models to plan, implement, and evaluate the educational interventions is recommended and emphasized for health researchers and educators.
